# Covid-19 testing, sick-pay and public health outbreak management of respiratory infections in care homes: three rapid reviews of the literature

**DOI:** 10.1093/pubmed/fdag025

**Published:** 2026-04-17

**Authors:** William Byrd, Nazak Salehi, Catherine Henderson

**Affiliations:** Care Policy and Evaluation Centre, London School of Economics and Political Science, London, WC2A 2AE, UK; Care Policy and Evaluation Centre, London School of Economics and Political Science, London, WC2A 2AE, UK; Care Policy and Evaluation Centre, London School of Economics and Political Science, London, WC2A 2AE, UK

**Keywords:** carers, COVID-19, health protection

## Abstract

**Background:**

Credible and costed plans for managing future outbreaks of Covid-19 and other respiratory infections depend on the availability of good quality evidence. Methods: Three rapid reviews (RRs) examined evidence on:

- Care home providers’ policy and practice on asymptomatic testing for Covid-19 and sickness absence, and/or unfilled shifts in the UK published since 2023 (RR1)
- Incidence of respiratory infections, and care home sick pay and/or shift backfill policies published internationally since 2023 (RR2)
- UK public health costs of managing care home outbreaks of respiratory infections (RR3).

Bibliographic database searches for each RR and supplementary grey literature searches of Google for RR1 and RR3.

**Results:**

RR1 included 1 study, RR2 none, and RR3, 1 report. RR1: a study of testing undertaken during an outbreak of Covid-19 in one care home. RR3: a report briefly described recommended inputs of one local authority’s public health service into managing outbreaks of respiratory infections in settings including care homes.

**Conclusion:**

The reviews found little-to-no recent evidence on care home providers’ policy and practice on asymptomatic Covid-19 testing, care home sick pay and/or shift backfill, and the incidence of Covid-19 and other respiratory infections, nor on costs of public health teams’ outbreak management.

## Introduction

Cost-effective strategies to prevent outbreaks of respiratory infections, including Covid-19, in care homes are needed. Three rapid reviews were undertaken to explore the availability of evidence of policy and practice on Covid-19 testing, sick pay and public heath outbreak management of respiratory infections in care homes. These were conducted to inform economic research within the VIVALDI-CT study.^1^

## Methods

Rapid reviewing typically streamlines standard systematic reviewing methods.^2^ Here we adapted the standard recommended methodology for a scoping review to meet our compressed timescales.^3^

Review questions:

- Rapid review 1 (RR1): What UK evidence has been published since 2023 about care home provider policy and practice relating to conducting asymptomatic testing for Covid-19 and sickness absence, and/or unfilled shifts?
- Rapid review 2 (RR2): What international evidence has been published since 2023 about the relationship between care home sick pay and/or shift backfill policy, and the incidence of Covid-19 and other respiratory infections?
- Rapid review 3 (RR3): What UK evidence has been published about health protection team and infection control team costs of managing outbreaks of Covid-19 and other respiratory infections in care homes?

### Bibliographic searches

Three bibliographic databases were searched for RR1 and RR2 (MEDLINE via Ovid, CINAHL via EBSCOhost, and Web of Science via Clarivate), and two were searched for RR3 (MEDLINE via Ovid, CINAHL via EBSCOhost), on 24 November 2023. The RR1 search strategy was structured around the key terms Covid-19 AND care homes AND (testing OR sick leave OR unfilled shifts). The RR2 search strategy used key terms: (Covid-19 OR respiratory infections) AND care homes AND (sick pay OR shift backfill). RR3 search strategy key terms were (Covid-19 OR respiratory infections) AND care homes AND (testing OR sick leave OR unfilled shifts OR outbreaks) AND economic terms from publicly available filters available for MEDLINE and CINAHL (full search strategies are described in S1). Results for RR1 and RR2 were limited to studies published since 2023, to focus on policy and practice in the period when the pandemic was in abatement. Records were de-duplicated in EndNote.^4^^,^^5^ Titles and abstracts were independently screened by two reviewers (WB and NS) on Rayyan.^6^ We used abstract screening tools^7^ for consistency across reviewers (S1). Conflicts were resolved in discussion with a third reviewer (CH).

### Grey literature

Google searches were undertaken between December 2023 and January 2024. A search strategy covering RR1 objectives was structured around the strings: respiratory infections AND care homes AND (testing OR sick leave OR unfilled shifts). The RR3 search strategy was structured around the strings: (respiratory outbreak OR infection response) AND care homes AND health protection. All results were limited to 2023. The objective was to find reports not arising from the bibliographic searches, so each search string was followed by a site filter for governmental, NHS, academic, and organizational related domains. One reviewer screened the first three pages of each set of results using abstract screening tools (Appendix 2).

## Results

**Figure 1:**
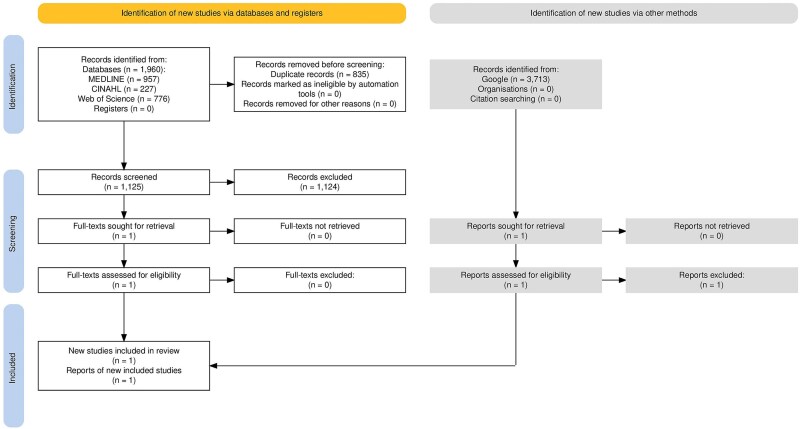
PRISMA flow diagram for RR1.

**Figure 2:**
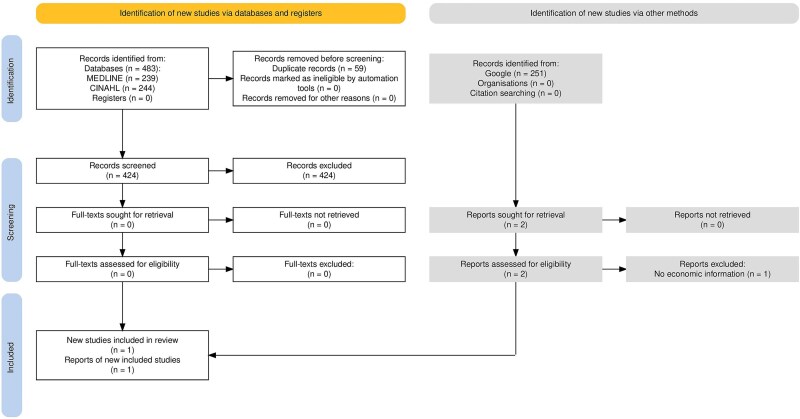
PRISMA flow diagram for RR3.

Flow diagrams are provided for RR1 (Fig. 1) and RR3 (Fig. 2),^8^ where one report was included in each review. For RR2, 483 records (424 after deduplication) were retrieved, and all were excluded at the title and abstract screening stage.

### Bibliographic searches

After title and abstract screening, one study was included for RR1, none in RR2, nor in RR3. After full text screening by two reviewers, RR1 included one study^9^ describing an outbreak of Covid-19 in one care home in the East of England, in August 2023. The paper covers the testing regime of giving PCR tests to all residents (*n* = 38) and all symptomatic or LFD-positive staff members (total staffing complement *n* = 66). Although regulations at this time did not require staff to test, all 11 symptomatic and 1 asymptomatic workers did voluntarily test (by PCR and/or LFD). All twelve tested positive. Staff members stayed off work for 5 days, or until they had no symptoms.

### Grey literature

After screening, no studies were included for RR1, while RR3 included one study.^10^ This report described the Operational Local Health Economy Outbreak Plan for Oldham. The document explains how major and locally confined smaller outbreaks will be recognized, which agencies will be involved, how local responses will be coordinated, and outlines the response activities involved in managing outbreaks. For care homes, if 2 or more residents of staff reported influenza-like illness (ILI)/ARI (including Covid-19), then Oldham Metropolitan Borough Council (OMBC) or UKHSA were to be alerted, and the information of affected staff/residents taken. Swabs would be obtained from symptomatic people (maximum 5), and the LA HPT would advise on infection prevention and control (IPC) measures.

## Discussion

### Main finding of this study

Evidence published since 2023 about the relationship between care home sick pay and/or shift backfill policy, and the incidence of Covid-19 and other respiratory infections was lacking. There is a near-absence of evidence on UK health protection and infection control teams’ costs of managing outbreaks of respiratory infections in care homes.

### What is already known on this topic

A rapid systematic review, spanning January 2020 to July 2022, found few published studies examining how policies incentivizing testing and financial support influence compliance with asymptomatic testing in care homes.^1^

### What this study adds

The three rapid reviews found little-to-no recent evidence. Credible and costed plans for managing future outbreaks of Covid-19 and other respiratory infections depend on the availability of good quality evidence.

### Limitations of this study

To streamline reviewing, three bibliographic databases were searched for RR1 and RR2, and two for RR3. A systematic review including more bibliographic databases may have identified more included studies.

## Supplementary Material

Supplementary_File_fdag025

## Data Availability

The data underlying this article are available in the article and in its online Supplementary Material*.*

## Notes

**William Byrd**, Research Assistant

**Nazak Salehi**, Research Officer

**Catherine Henderson**, Assistant Professorial Research Fellow
