# Supplementary material for: Covid-19 testing, sick-pay and public health outbreak management of respiratory infections in care homes: three rapid reviews of the literature
Source: J Public Health (Oxf). 2026 Apr 17;48(2):539–42. doi: 10.1093/pubmed/fdag025 (PMC13223584; doi:10.1093/pubmed/fdag025)
Supplement: Supplementary_File_fdag025 [file supplementary_file_fdag025.docx]

**Supplementary file**

1. **Results of bibliographic searches**

| **#** | **Search term**  **(Search for MEDLINE via Ovid)** | **No. of hits** |
| --- | --- | --- |
| 1 | SARS-CoV-2/ or COVID-19/ | 253017 |
| 2 | (corona* adj1 (virus* or viral*)).ti,ab,kw,kf. | 6540 |
| 3 | (covid* or coronavirus* or 2019nCoV* or 19nCoV* or "2019 novel*" or Ncov* or "n-cov" or "SARS-CoV-2*" or "SARSCoV-2*" or SARSCoV2* or "SARS-CoV2*" or "severe acute respiratory syndrome*" or pandemic* or longcovid* or postcovid* or postcoronavirus* or postsars*).ti,ab,kw,kf. | 441530 |
| 4 | or/1-3 | 447073 |
| 5 | ((intermediate or “long term” or longterm or institution* or day or extended or respite) adj3 care).ti,ab,kw,kf | 62310 |
| 6 | Residential Facilities/ or exp Nursing Homes/ or Respite Care/ or Long-Term Care/ or Housing for the Elderly/ or Geriatric Nursing/ or Adult Day Care Centres/ or Assisted Living Facilities/ or Homes for the Aged/ | 91452 |
| 7 | (("old age" or "old* people*" or “old* person*” or “old* adult*” or aged or geriatric* or retirement or nursing or care or resident* or long-term or longterm or senior* or aging or ageing or elder*) adj3 (home? or institution* or facility or facilities or hous* or center* or centre* or unit or units or establishment*)).ti,ab,kw,kf. | 414713 |
| 8 | or/5-7 | 480669 |
| 9 | COVID-19 Testing/ | 9556 |
| 10 | ((asymptomatic or covid* or coronavirus* or “SARS-CoV-2” or “SARSCoV-2*” or SARSCoV2* or “SARS-Cov2*” or PCR or rapid or “lateral flow”) adj3 (test* or swab* or kit* or assay* or measure* or immunoassay* or detect* or screen* or diagnos*)).ti,ab,kw,kf. | 267443 |
| 11 | Sick Leave/ or Occupational Health/ | 42880 |
| 12 | ((sick* or ill* or unwell or authori#ed or excused or permitted or unpaid or medical*) adj3 (leave or absence* or “time off” or day*)).ti,ab,kw,kf. | 20409 |
| 13 | (((employee* or staff or work* or shift*) adj3 (shortage* or gap* or missing or absence*)) or “short-staffed”).ti,ab,kw,kf. | 10818 |
| 14 | or/9-13 | 337132 |
| 15 | 4 and 8 and 14 | 5078 |
| 16 | limit 15 to yr="2023 -Current" | 957 |
| 17 | exp Animals/ not Humans/ | 5173743 |
| 18 | 16 not 17 | 957 |

*Table I: First RR: Covid-19 AND Care Homes AND (testing OR sick leave OR unfilled shifts) – No. of hits as of 24/11/23*

| **#** | **Search term**  **(Search for CINAHL via EBSCOhost)** | **No. of hits** |
| --- | --- | --- |
| 1 | (MH “SARS-CoV-2”) or (MH “COVID-19+”) | 46046 |
| 2 | TI (corona* N1 (virus* or viral*)) or AB (corona* N1 (virus* or viral*)) | 903 |
| 3 | TI (covid* or coronavirus* or 2019nCoV* or 19nCoV* or "2019 novel*" or Ncov* or "n-cov" or "SARS-CoV-2*" or "SARSCoV-2*" or SARSCoV2* or "SARS-CoV2*" or "severe acute respiratory syndrome*" or pandemic* or longcovid* or postcovid* or postcoronavirus* or postsars*) or AB (covid* or coronavirus* or 2019nCoV* or 19nCoV* or "2019 novel*" or Ncov* or "n-cov" or "SARS-CoV-2*" or "SARSCoV-2*" or SARSCoV2* or "SARS-CoV2*" or "severe acute respiratory syndrome*" or pandemic* or longcovid* or postcovid* or postcoronavirus* or postsars*) | 150331 |
| 4 | S1 or S2 or S3 | 155085 |
| 5 | TI ((intermediate or “long term” or longterm or institution* or day or extended or respite) N2 care) or AB ((intermediate or “long term” or longterm or institution* or day) N2 care or extended or respite) | 38621 |
| 6 | (MH “Residential Facilities”) or (MH “Nursing Homes”) or (MH “Respite Care”) or (MH “Long-Term Care”) or (MH “Housing for Older Persons”) or (MH “Gerontologic Nursing”) | 48542 |
| 7 | TI (("old age" or "old* people*" or “old* person*” or “old* adult*” or aged or geriatric* or retirement or nursing or care or resident* or long-term or longterm or senior* or aging or ageing or elder*) N2 (home# or institution* or facility or facilities or hous* or center* or centre* or unit or units or establishment*)) or AB (("old age" or "old* people*" or “old* person*” or “old* adult*” or aged or geriatric* or retirement or nursing or care or resident* or long-term or longterm or senior* or aging or ageing or elder*) N2 (home# or institution* or facility or facilities or hous* or center* or centre* or unit or units or establishment*)) | 217221 |
| 8 | S5 or S6 or S7 | 257486 |
| 9 | (MH “COVID-19 Testing”) | 2422 |
| 10 | TI ((asymptomatic or covid* or coronavirus* or “SARS-CoV-2” or “SARSCoV-2*” or SARSCoV2* or “SARS-Cov2*” or PCR or rapid or “lateral flow”) N2 (test* or swab* or kit* or assay* or measure* or immunoassay* or detect* or screen* or diagnos*)) or AB ((asymptomatic or covid* or coronavirus* or “SARS-CoV-2” or “SARSCoV-2*” or SARSCoV2* or “SARS-Cov2*” or PCR or rapid or “lateral flow”) N2 (test* or swab* or kit* or assay* or measure* or immunoassay* or detect* or screen* or diagnos*)) | 32856 |
| 11 | (MH “Sick Leave”) or (MH “Family and Medical Leave”) or (MH “Occupational Health”) | 35989 |
| 12 | TI ((sick* or ill* or unwell or authori?ed or excused or permitted or unpaid or medical*) N2 (leave or absence* or “time off” or day*)) or AB ((sick* or ill* or unwell or authori?ed or excused or permitted or unpaid or medical*) N2 (leave or absence* or “time off” or day*)) | 8346 |
| 13 | TI (((employee* or staff or work* or shift*) N2 (shortage* or gap* or missing or absence*)) or “short-staffed”) or AB (((employee* or staff or work* or shift*) N2 (shortage* or gap* or missing or absence*)) or “short-staffed”) | 6074 |
| 14 | S9 or S10 or S11 or S12 or S13 | 80022 |
| 15 | S4 and S8 and S14 | 1707 |
| 16 | (MH “Animals+” or “Animal Studies” or TI “animal model*”) not MH “Human” | 218386 |
| 17 | S15 not S16 | 1707 |
| 18 | Limiters – Published Date: 20230101-20240101 | 227 |

*Table II: First RR: Covid-19 AND Care Homes AND (testing OR sick leave OR unfilled shifts) – No. of hits as of 24/11/23*

| **#** | **Search term**  **(Search for Web of Science via Clarivate)** | **No. of hits** |
| --- | --- | --- |
| 1 | TS = (corona* NEAR/1 (virus* or viral*)) or (covid* or coronavirus* or 2019nCoV* or 19nCoV* or "2019 novel*" or Ncov* or "n-cov" or "SARS-CoV-2*" or "SARSCoV-2*" or SARSCoV2* or "SARS-CoV2*" or "severe acute respiratory syndrome*" or pandemic* or longcovid* or postcovid* or postcoronavirus* or postsars*) | 572079 |
| 2 | TS = ((intermediate or “long term” or longterm or institution* or day or extended or respite) NEAR/2 care) | 71036 |
| 3 | TS = (("old age" or "old* people*" or “old* person*” or “old* adult*” or aged or geriatric* or retirement or nursing or care or resident* or long-term or longterm or senior* or aging or ageing or elder*) NEAR/2 (home? or institution* or facility or facilities or hous* or center* or centre* or unit or units or establishment*)) | 399658 |
| 4 | #2 or #3 | 432525 |
| 5 | TS = ((asymptomatic or covid* or coronavirus* or “SARS-CoV-2” or “SARSCoV-2*” or SARSCoV2* or “SARS-Cov2*” or PCR or rapid or “lateral flow”) NEAR/2 (test* or swab* or kit* or assay* or measure* or immunoassay* or detect* or screen* or diagnos*)) | 295421 |
| 6 | TS = ((sick* or ill* or unwell or authorised or authorized or excused or permitted or unpaid or medical*) NEAR/2 (leave or absence* or “time off” or day*)). | 25716 |
| 7 | TS = (((employee* or staff or work* or shift*) NEAR/2 (shortage* or gap* or missing or absence*)) or “short-staffed”). | 21155 |
| 8 | #5 or #6 or #7 | 340334 |
| 9 | #1 and #4 and #8 | 4469 |
| 10 | #9 and (PY=2023) | 776 |

*Table III: First RR: Covid-19 AND Care Homes AND (testing OR sick leave OR unfilled shifts) – No. of hits as of 24/11/23*

| **#** | **Search term**  **(Search for MEDLINE via Ovid)** | **No. of hits** |
| --- | --- | --- |
| 1 | SARS-CoV-2/ or COVID-19/ | 253017 |
| 2 | (corona* adj1 (virus* or viral*)).ti,ab,kw,kf. | 6540 |
| 3 | (covid* or coronavirus* or 2019nCoV* or 19nCoV* or "2019 novel*" or Ncov* or "n-cov" or "SARS-CoV-2*" or "SARSCoV-2*" or SARSCoV2* or "SARS-CoV2*" or "severe acute respiratory syndrome*" or pandemic* or longcovid* or postcovid* or postcoronavirus* or postsars*).ti,ab,kw,kf. | 441530 |
| 4 | exp Respiratory Tract Infections/ or exp Influenza, Human/ or Respiratory Syncytial Virus, Human/ | 633171 |
| 5 | (flu or influenza* or pneumonia* or rsv or (respiratory adj1 (virus* or viral* or infectio*))).ti,ab,kw,kf | 388255 |
| 6 | or/1-5 | 1028295 |
| 7 | ((intermediate or “long term” or longterm or institution* or day or extended or respite) adj3 care).ti,ab,kw,kf | 62310 |
| 8 | Residential Facilities/ or exp Nursing Homes/ or Respite Care/ or Long-Term Care/ or Housing for the Elderly/ or Geriatric Nursing/ or Adult Day Care Centres/ or Assisted Living Facilities/ or Homes for the Aged/ | 91452 |
| 9 | (("old age" or "old* people*" or “old* person*” or “old* adult*” or aged or geriatric* or retirement or nursing or care or resident* or long-term or longterm or senior* or aging or ageing or elder*) adj3 (home? or institution* or facility or facilities or hous* or center* or centre* or unit or units or establishment*)).ti,ab,kw,kf. | 414713 |
| 10 | or/7-9 | 480669 |
| 11 | Sick Leave/ or Occupational Health/ | 42880 |
| 12 | ((sick* or ill* or unwell or authori#ed or excused or permitted or unpaid or medical*) adj3 (leave or absence* or “time off” or day*)).ti,ab,kw,kf. | 20409 |
| 13 | ((sick* or ill* or absence*) adj3 (pay* or benefit* or money or compensat* or insur* or allow*)).ti,ab,kw,kf. | 8761 |
| 14 | (((employee* or staff or work* or shift*) adj3 (shortage* or gap* or missing or absence*)) or “short-staffed”).ti,ab,kw,kf. | 10818 |
| 15 | (backfill* or overtime or agenc* or promotion* or cover* or “on-call” or “under* shift*” or locum*).ti,ab,kw,kf. | 742704 |
| 16 | or/11-15 | 812512 |
| 17 | 6 and 10 and 16 | 2666 |
| 18 | limit 17 to yr="2023 -Current" | 423 |
| 19 | exp Animals/ not Humans/ | 5173743 |
| 20 | 18 not 19 | 423 |

*Table IV: Second RR: (Covid-19 OR respiratory infections) AND Care homes AND (sick pay OR shift backfill) – No. of hits as of 24/11/23*

| **#** | **Search term**  **(Search for CINAHL via EBSCOhost)** | **No. of hits** |
| --- | --- | --- |
| 1 | (MH “SARS-CoV-2”) or (MH “COVID-19+”) | 46046 |
| 2 | TI (corona* N1 (virus* or viral*)) or AB (corona* N1 (virus* or viral*)) | 903 |
| 3 | TI (covid* or coronavirus* or 2019nCoV* or 19nCoV* or "2019 novel*" or Ncov* or "n-cov" or "SARS-CoV-2*" or "SARSCoV-2*" or SARSCoV2* or "SARS-CoV2*" or "severe acute respiratory syndrome*" or pandemic* or longcovid* or postcovid* or postcoronavirus* or postsars*) or AB (covid* or coronavirus* or 2019nCoV* or 19nCoV* or "2019 novel*" or Ncov* or "n-cov" or "SARS-CoV-2*" or "SARSCoV-2*" or SARSCoV2* or "SARS-CoV2*" or "severe acute respiratory syndrome*" or pandemic* or longcovid* or postcovid* or postcoronavirus* or postsars*) | 150331 |
| 4 | (MH “Influenza”) or (MH “Pneumonia”) or (MH “Respiratory Tract Infections) or (MH “Respiratory Syncytial Viruses”) or (MH “Respiratory Syncytial Virus Infections”) | 34605 |
| 5 | TI (flu or influenza* or pneumonia* or rsv or (respiratory N1 (virus* or viral* or infectio*))) or AB (flu or influenza* or pneumonia* or rsv or (respiratory N1 (virus* or viral* or infectio*))) | 80859 |
| 6 | S1 or S2 or S3 or S4 or S5 | 234539 |
| 7 | TI ((intermediate or “long term” or longterm or institution* or day or extended or respite) N2 care) or AB ((intermediate or “long term” or longterm or institution* or day or extended or respite) N2 care) | 38621 |
| 8 | (MH “Residential Facilities”) or (MH “Nursing Homes”) or (MH “Respite Care”) or (MH “Long-Term Care”) or (MH “Housing for Older Persons”) or (MH “Gerontologic Nursing”) | 48542 |
| 9 | TI (("old age" or "old* people*" or “old* person*” or “old* adult*” or aged or geriatric* or retirement or nursing or care or resident* or long-term or longterm or senior* or aging or ageing or elder*) N2 (home# or institution* or facility or facilities or hous* or center* or centre* or unit or units or establishment*)) or AB (("old age" or "old* people*" or “old* person*” or “old* adult*” or aged or geriatric* or retirement or nursing or care or resident* or long-term or longterm or senior* or aging or ageing or elder*) N2 (home# or institution* or facility or facilities or hous* or center* or centre* or unit or units or establishment*)) | 217221 |
| 10 | S7 or S8 or S9 | 257486 |
| 11 | (MH “Sick Leave”) or (MH “Family and Medical Leave”) or (MH “Occupational Health”) | 35989 |
| 12 | TI ((sick* or ill* or unwell or authori?ed or excused or permitted or unpaid or medical*) N2 (leave or absence* or “time off” or day*)) or AB ((sick* or ill* or unwell or authori?ed or excused or permitted or unpaid or medical*) N2 (leave or absence* or “time off” or day*)) | 8346 |
| 13 | TI ((sick* or ill* or absence*) N2 (pay* or benefit* or money or compensat* or insur* or allow*)) or AB ((sick* or ill* or absence*) N2 (pay* or benefit* or money or compensat* or insur* or allow*)) | 2619 |
| 14 | TI (((employee* or staff or work* or shift*) N2 (shortage* or gap* or missing or absence*)) or “short-staffed”) or AB (((employee* or staff or work* or shift*) N2 (shortage* or gap* or missing or absence*)) or “short-staffed”) | 6074 |
| 15 | TI (backfill* or overtime or agenc* or promotion* or cover* or “on-call” or “under* shift*” or locum*) or AB (backfill* or overtime or agenc* or promotion* or cover* or “on-call” or “under* shift*” or locum*) | 268023 |
| 16 | S11 or S12 or S13 or S14 or S15 | 312920 |
| 17 | S6 and S10 and S16 | 1383 |
| 18 | (MH “Animals+” or “Animal Studies” or TI “animal model*”) not MH “Human” | 218386 |
| 19 | S17 not S18 | 1379 |
| 20 | Limiters – Published Date: 20230101-20240101 | 159 |

*Table V: Second RR: (Covid-19 OR respiratory infections) AND Care homes AND (sick pay OR shift backfill) – No. of hits as of 24/11/23*

| **#** | **Search term**  **(Search for Web of Science via Clarivate)** | **No. of hits** |
| --- | --- | --- |
| 1 | TS = (corona* NEAR/1 (virus* or viral*)) or (covid* or coronavirus* or 2019nCoV* or 19nCoV* or "2019 novel*" or Ncov* or "n-cov" or "SARS-CoV-2*" or "SARSCoV-2*" or SARSCoV2* or "SARS-CoV2*" or "severe acute respiratory syndrome*" or pandemic* or longcovid* or postcovid* or postcoronavirus* or postsars*) | 572079 |
| 2 | TS = (flu or influenza* or pneumonia* or rsv or (respiratory NEAR/1 (virus* or viral* or infectio*))) | 467534 |
| 3 | #1 or #2 | 974872 |
| 4 | TS = ((intermediate or “long term” or longterm or institution* or day or extended or respite) NEAR/2 care) | 71036 |
| 5 | TS = (("old age" or "old* people*" or “old* person*” or “old* adult*” or aged or geriatric* or retirement or nursing or care or resident* or long-term or longterm or senior* or aging or ageing or elder*) NEAR/2 (home? or institution* or facility or facilities or hous* or center* or centre* or unit or units or establishment*)) | 399658 |
| 6 | #4 or #5 | 432525 |
| 7 | TS = ((sick* or ill* or unwell or authorised or authorized or excused or permitted or unpaid or medical*) NEAR/2 (leave or absence* or “time off” or day*)). | 25716 |
| 8 | TS = ((sick* or ill* or absence*) NEAR/2 (pay* or benefit* or money or compensat* or insur* or allow*)). | 16803 |
| 9 | TS = (((employee* or staff or work* or shift*) NEAR/2 (shortage* or gap* or missing or absence*)) or “short-staffed”). | 21155 |
| 10 | TS = (backfill* or overtime or agenc* or promotion* or cover* or “on-call” or “under* shift*” or locum*) | 1840399 |
| 11 | #7 or #8 or #9 or #10 | 1897673 |
| 12 | #3 and #6 and #11 | 2727 |
| 13 | #12 and (PY=2023) | 386 |

*Table VI: Second RR: (Covid-19 OR respiratory infections) AND Care homes AND (sick pay OR shift backfill) – No. of hits as of 24/11/23*

| **#** | **Search term**  **(Search for MEDLINE via Ovid)** | **No. of hits** |
| --- | --- | --- |
| 1 | SARS-CoV-2/ or COVID-19/ | 253017 |
| 2 | (corona* adj1 (virus* or viral*)).ti,ab,kw,kf. | 6540 |
| 3 | (covid* or coronavirus* or 2019nCoV* or 19nCoV* or "2019 novel*" or Ncov* or "n-cov" or "SARS-CoV-2*" or "SARSCoV-2*" or SARSCoV2* or "SARS-CoV2*" or "severe acute respiratory syndrome*" or pandemic* or longcovid* or postcovid* or postcoronavirus* or postsars*).ti,ab,kw,kf. | 441530 |
| 4 | exp Respiratory Tract Infections/ or exp Influenza, Human/ or Respiratory Syncytial Virus, Human/ | 635731 |
| 5 | (flu or influenza* or pneumonia* or rsv or (respiratory adj1 (virus* or viral* or infectio*))).ti,ab,kw,kf | 388255 |
| 6 | or/1-5 | 1028739 |
| 7 | ((intermediate or “long term” or longterm or institution* or day or extended or respite) adj3 care).ti,ab,kw,kf | 62310 |
| 8 | Residential Facilities/ or exp Nursing Homes/ or Respite Care/ or Long-Term Care/ or Housing for the Elderly/ or Geriatric Nursing/ or Adult Day Care Centres/ or Assisted Living Facilities/ or Homes for the Aged/ | 91452 |
| 9 | (("old age" or "old* people*" or “old* person*” or “old* adult*” or aged or geriatric* or retirement or nursing or care or resident* or long-term or longterm or senior* or aging or ageing or elder*) adj3 (home? or institution* or facility or facilities or hous* or center* or centre* or unit or units or establishment*)).ti,ab,kw,kf. | 414713 |
| 10 | or/7-9 | 480669 |
| 11 | COVID-19 Testing/ | 9556 |
| 12 | ((symptomatic or asymptomatic or covid* or coronavirus* or “SARS-CoV-2” or “SARSCoV-2*” or SARSCoV2* or “SARS-Cov2*” or PCR or rapid or “lateral flow”) adj3 (test* or swab* or kit* or assay* or measure* or immunoassay* or detect* or screen* or diagnos*)).ti,ab,kw,kf. | 274901 |
| 13 | Sick Leave/ or Occupational Health/ | 42880 |
| 14 | ((sick* or ill* or unwell or authori#ed or excused or permitted or unpaid or medical*) adj3 (leave or absence* or “time off” or day*)).ti,ab,kw,kf. | 20409 |
| 15 | (((employee* or staff or work* or shift*) adj3 (shortage* or gap* or missing or absence*)) or “short-staffed”).ti,ab,kw,kf. | 10818 |
| 16 | Disease Outbreaks/ | 92172 |
| 17 | ((outbreak* or inciden* or case* or infection* or “public health”) adj3 response).ti,ab,kw,kf | 40500 |
| 18 | or/11-17 | 383952 |
| 19 | Economics/ or exp "Costs and Cost Analysis"/ or Economics, Nursing/ or Economics, Medical/ or Economics, Pharmaceutical/ or exp Economics, Hospital/ or Economics, Dental/ or exp "Fees and Charges"/ or exp Budgets/ or exp Models, Economic/ or Markov Chains/ or exp Decision Theory/ or Monte Carlo Method/ | 399516 |
| 20 | (budget*).ti,ab,kf. | 36926 |
| 21 | (economic* or cost or costs or costly or costing or price or prices or pricing or pharmacoeconomic* or pharmaco-economic* or expenditure or expenditures or expense or expenses or financial or finance or finances or financed).ti,kf. | 287990 |
| 22 | (economic* or cost or costs or costly or costing or price or prices or pricing or pharmacoeconomic* or pharmaco-economic* or expenditure or expenditures or expense or expenses or financial or finance or finances or financed).ab. /freq=2 | 392654 |
| 23 | (cost* adj2 (effective* or utilit* or benefit* or minimi* or analy* or outcome or outcomes)).ab,kf. | 216173 |
| 24 | (value adj2 (money or monetary)).ti,ab,kf. | 3125 |
| 25 | economic model*.ab,kf. | 4335 |
| 26 | markov.ti,ab,kf. | 29880 |
| 27 | monte carlo.ti,ab,kf. | 61505 |
| 28 | (decision* adj2 (tree* or analy* or model*)).ti,ab,kf. | 40011 |
| 29 | or/19-28 | 919579 |
| 30 | 6 and 10 and 18 and 29 | 240 |
| 31 | exp Animals/ not Humans/ | 5173743 |
| 32 | 30 not 31 | 239 |

*Table VII: Third RR (Covid-19 OR respiratory infections) AND Care homes AND (testing OR sick leave OR unfilled shifts OR outbreaks) AND economic filter – No. hits as of 24/11/23*

| **#** | **Search term**  **(Search for CINAHL via EBSCOhost)** | **No. of hits** |
| --- | --- | --- |
| 1 | (MH “SARS-CoV-2”) or (MH “COVID-19+”) | 46046 |
| 2 | TI (corona* N1 (virus* or viral*)) or AB (corona* N1 (virus* or viral*)) | 903 |
| 3 | TI (covid* or coronavirus* or 2019nCoV* or 19nCoV* or "2019 novel*" or Ncov* or "n-cov" or "SARS-CoV-2*" or "SARSCoV-2*" or SARSCoV2* or "SARS-CoV2*" or "severe acute respiratory syndrome*" or pandemic* or longcovid* or postcovid* or postcoronavirus* or postsars*) or AB (covid* or coronavirus* or 2019nCoV* or 19nCoV* or "2019 novel*" or Ncov* or "n-cov" or "SARS-CoV-2*" or "SARSCoV-2*" or SARSCoV2* or "SARS-CoV2*" or "severe acute respiratory syndrome*" or pandemic* or longcovid* or postcovid* or postcoronavirus* or postsars*) | 150331 |
| 4 | (MH “Influenza”) or (MH “Pneumonia”) or (MH “Respiratory Tract Infections) or (MH “Respiratory Syncytial Viruses”) or (MH “Respiratory Syncytial Virus Infections”) | 23224 |
| 5 | TI (flu or influenza* or pneumonia* or rsv or (respiratory N1 (virus* or viral* or infectio*))) or AB (flu or influenza* or pneumonia* or rsv or (respiratory N1 (virus* or viral* or infectio*))) | 80859 |
| 6 | S1 or S2 or S3 or S4 or S5 | 230572 |
| 7 | TI ((intermediate or “long term” or longterm or institution* or day or extended or respite) N2 care) or AB ((intermediate or “long term” or longterm or institution* or day) N2 care or extended or respite) | 77884 |
| 8 | (MH “Residential Facilities”) or (MH “Nursing Homes”) or (MH “Respite Care”) or (MH “Long-Term Care”) or (MH “Housing for Older Persons”) or (MH “Gerontologic Nursing”) | 48542 |
| 9 | TI (("old age" or "old* people*" or “old* person*” or “old* adult*” or aged or geriatric* or retirement or nursing or care or resident* or long-term or longterm or senior* or aging or ageing or elder*) N2 (home# or institution* or facility or facilities or hous* or center* or centre* or unit or units or establishment*)) or AB (("old age" or "old* people*" or “old* person*” or “old* adult*” or aged or geriatric* or retirement or nursing or care or resident* or long-term or longterm or senior* or aging or ageing or elder*) N2 (home# or institution* or facility or facilities or hous* or center* or centre* or unit or units or establishment*)) | 217221 |
| 10 | S7 or S8 or S9 | 294732 |
| 11 | (MH “COVID-19 Testing”) | 2422 |
| 12 | TI ((asymptomatic or covid* or coronavirus* or “SARS-CoV-2” or “SARSCoV-2*” or SARSCoV2* or “SARS-Cov2*” or PCR or rapid or “lateral flow”) N2 (test* or swab* or kit* or assay* or measure* or immunoassay* or detect* or screen* or diagnos*)) or AB ((asymptomatic or covid* or coronavirus* or “SARS-CoV-2” or “SARSCoV-2*” or SARSCoV2* or “SARS-Cov2*” or PCR or rapid or “lateral flow”) N2 (test* or swab* or kit* or assay* or measure* or immunoassay* or detect* or screen* or diagnos*)) | 32856 |
| 13 | (MH “Sick Leave”) or (MH “Family and Medical Leave”) or (MH “Occupational Health”) | 35989 |
| 14 | TI ((sick* or ill* or unwell or authori?ed or excused or permitted or unpaid or medical*) N2 (leave or absence* or “time off” or day*)) or AB ((sick* or ill* or unwell or authori?ed or excused or permitted or unpaid or medical*) N2 (leave or absence* or “time off” or day*)) | 8346 |
| 15 | TI (((employee* or staff or work* or shift*) N2 (shortage* or gap* or missing or absence*)) or “short-staffed”) or AB (((employee* or staff or work* or shift*) N2 (shortage* or gap* or missing or absence*)) or “short-staffed”) | 6074 |
| 16 | (MH “Disease Outbreaks”) | 42157 |
| 17 | TI ((outbreak* or inciden* or case* or infection* or “public health”) N2 (response)) or AB ((outbreak* or inciden* or case* or infection* or “public health”) N2 (response)) | 7465 |
| 18 | S11 or S12 or S13 or S14 or S15 or S16 or S17 | 125972 |
| 19 | MH "Economics" OR MH "Costs and Cost Analysis+" OR MH "Economic Aspects of Illness" OR MH "Resource Allocation+" OR MH "Economic Value of Life" OR MH "Economics, Pharmaceutical" OR MH "Economics, Dental" OR MH "Fees and Charges+" OR MH "Budgets" OR MH "Decision Trees" OR TI budget* OR TI ( economic* OR cost OR costs OR costly OR costing OR price OR prices OR pricing OR pharmacoeconomic* OR "pharmaco-economic*" OR expenditure OR expenditures OR expense OR expenses OR financial OR finance OR finances OR financed ) OR TI ( cost* N2 (effective* OR utilit* OR benefit* OR minimi* OR analy* OR outcome OR outcomes) ) OR TI ( value N2 (money OR monetary) ) OR TI ( markov OR monte carlo ) OR TI ( decision* N2 (tree* OR analy* OR model*) ) OR AB budget* OR AB ( economic* OR cost OR costs OR costly OR costing OR price OR prices OR pricing OR pharmacoeconomic* OR "pharmaco-economic*" OR expenditure OR expenditures OR expense OR expenses OR financial OR finance OR finances OR financed ) OR AB ( cost* N2 (effective* OR utilit* OR benefit* OR minimi* OR analy* OR outcome OR outcomes) ) OR AB ( value N2 (money OR monetary) ) OR AB ( markov OR monte carlo ) OR AB ( decision* N2 (tree* OR analy* OR model*) ) | 468591 |
| 20 | S6 and S10 and S18 and S19 | 247 |
| 21 | (MH “Animals+” or “Animal Studies” or TI “animal model*”) not MH “Human” | 218386 |
| 22 | S20 not S21 | 244 |

*Table VIII: Third RR (Covid-19 OR respiratory infections) AND Care homes AND (testing OR sick leave OR unfilled shifts OR outbreaks) AND economic filter – No. hits as of 24/11/23*

1. **Abstract screening tools**

| **Rapid Review 1**  Abstract screening tool   1. **Does the study report on research published in English?** 2. **Does the study report on original research?** E.g., not reviews or opinion pieces 3. **Does the study report on research undertaken in the UK?** 4. **Does the study report on the period since January 2023?** 5. **Does the study report on institutional care?** including all non-acute residential and nursing facilities that house people with some form of LTC needs (e.g., nursing homes (including both skilled nursing facilities and intermediate care facilities) and residential facilities (such as assisted living facilities, group homes, and other homes for the elderly and others in need of LTC services). 6. **Does the study report on:** 7. **asymptomatic testing** 8. **sickness absence** 9. **and/or unfilled shifts** |
| --- |

*Table IX: First RR abstract screening tool*

| **Rapid Review 2**  Abstract screening tool   1. **Does the study report on original research?** E.g., not reviews or opinion pieces 2. **Does the study report on the period since January 2023?** 3. **Does the study report on institutional care?** including all non-acute residential and nursing facilities that house people with some form of LTC needs (e.g., nursing homes (including both skilled nursing facilities and intermediate care facilities) and residential facilities (such as assisted living facilities, group homes, and other homes for the elderly and others in need of LTC services). 4. **Does the study report on policy for:** 5. **sick pay** 6. **and/or shift backfill** 7. **Does the study report outcomes of policy on the incidence of Covid-19 and/or other respiratory infections?** |
| --- |

*Table X: Second RR abstract screening tool*

| **Rapid Review 3**  Abstract screening tool   1. **Does the study report on research published in English?** 2. **Does the study report on original research?** E.g., not reviews or opinion pieces 3. **Does the study report on research undertaken in the UK?** 4. **Does the study report on institutional care?** including all non-acute residential and nursing facilities that house people with some form of LTC needs (e.g., nursing homes (including both skilled nursing facilities and intermediate care facilities) and residential facilities (such as assisted living facilities, group homes, and other homes for the elderly and others in need of LTC services). 5. **Does the study report on outbreaks of Covid-19 and other respiratory infections?** 6. **Does the study report on economic evaluations of processes involved in managing outbreaks?** including cost studies and healthcare utilisation studies 7. **Does the study report on the actions of health protection teams and infection control teams?** |
| --- |

Table XI: Third RR abstract screening tool
